# Maintaining neural stem cell identity in the brain

**DOI:** 10.7554/eLife.05000

**Published:** 2014-11-06

**Authors:** Yanrui Jiang, Heinrich Reichert

**Affiliations:** 1 **Yanrui Jiang** is in the Biozentrum, University of Basel, Basel, Switzerlandyanrui.jiang@unibas.ch; 2 **Heinrich Reichert** is in the Biozentrum, University of Basel, Basel, Switzerlandheinrich.reichert@unibas.ch

**Keywords:** neuroblast, intermediate neural progenitor, *trithorax*, *buttonhead*, neural stem cells, *pointed*, *D. melanogaster*

## Abstract

In the developing fruit fly brain, a protein called Trithorax increases the number of neural cells produced from a single stem cell, in part by regulating the transcription of the target genes *buttonhead* and *pointed*.

**Related research articles** Komori H, Xiao Q, Janssens DH, Dou Y, Lee CY. 2014. Trithorax maintains the functional heterogeneity of neural stem cells through the transcription factor Buttonhead. *eLife* **3**:e03502. doi: 10.7554/eLife.03502

Xie Y, Li X, Zhang X, Mei S, Li H, Urso A, Zhu S. 2014. The Drosophila Sp8 transcription factor Buttonhead prevents premature differentiation of intermediate neural progenitors. *eLife* **3**:e03596. doi: 10.7554/eLife.03596

**Image** Reducing the amount of a protein called Buttonhead from Type II neuroblasts in the fruit fly brain causes the cells to behave more like Type I neuroblasts

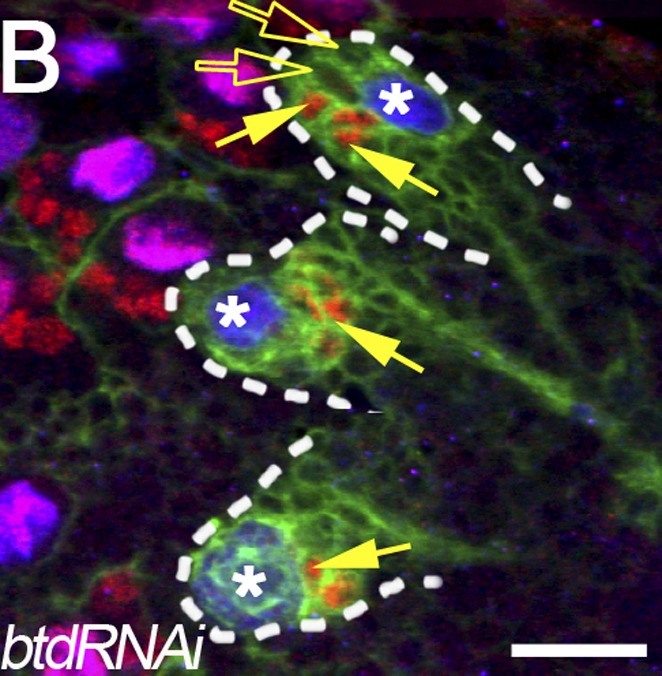

Stem cells are cells that have the ability to divide and produce both new stem cells (a process called self-renewal) and specific types of cell (a process called differentiation). There are many different types of stem cell. The brain of the fruit fly *Drosophila*, for example, contains two different types of neural stem cell. Type I neuroblasts divide to produce a new type I neuroblast and a cell called a ganglion mother cell. The new neuroblast can undergo several more rounds of division, but the ganglion mother cell can only divide once, to produce specific cell types (either neurons or glia cells). Type II neuroblasts, on the other hand, divide to produce a new type II neuroblast and a cell called an intermediate neural progenitor. Each intermediate neural progenitor cell can undergo several rounds of proliferation, each of which produces a ganglion mother cell (Homem and Knoblich, 2012). This ‘amplifies’ the number of neurons and glia cells that are produced.

Controlling the proliferation and differentiation of intermediate neural progenitors as the brain develops is crucial because these cells can also undergo a developmental reversal that results in them becoming neuroblast-like cells. These can then overproliferate and form malignant tumours in the brain. Recent studies in *Drosophila* have identified several regulatory proteins that prevent this developmental reversal and restrict the proliferation of intermediate neural progenitors. These include the post-transcriptional regulator Brain tumor (Bello et al., 2006; Betschinger et al., 2006; Lee et al., 2006), the Notch signaling pathway component Numb (Wang et al., 2007), the *Drosophila* SWI/SNF chromatin remodeling complex (Eroglu et al., 2014), and the zinc-finger transcription factor Earmuff (Weng et al., 2010). These proteins also work together to restrict proliferation in type II neuroblasts and the cells that they generate (Figure 1).

**Figure 1.**
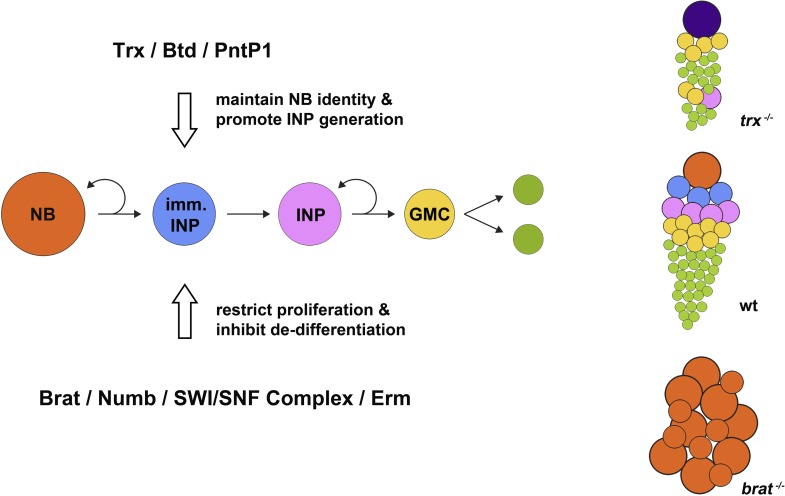
Maintaining neural stem cell identity in the *Drosophila* brain. A type II neuroblast (NB; orange circles) divides to self-renew and to give rise to an immature intermediate neural progenitor cell (imm. INP; blue circles), which becomes a mature INP (pink) that undergoes several further rounds of self-renewing proliferation. Each round generates a ganglion mother cell (GMC; yellow), each of which gives rise to two differentiated neural cells (green). During neuroblast proliferation, several proteins, including Brat, Numb, SWI/SNF complex, and Erm, restrict the ability of INPs to proliferate and inhibit the de-differentiation of immature INPs. In mutants that lack the *brain tumor* gene (*brat*^*−/−*^; bottom right), immature INPs revert to neuroblast-like cells that overproliferate and can form brain tumours. On the other hand, as shown by Komori et al. and Xie et al., Trithorax (Trx) and its direct targets Buttonhead (Btd) and Pointed P1 (PntP1) act to maintain the identity of type II neuroblasts and promote the generation of INPs. Hence, in mutants lacking the *trithorax* gene (*trx*^*−/−*^ mutants; top right), INPs are nearly completely lost compared with wild type (wt; centre right), and type II neuroblasts are transformed into type I neuroblasts (purple) that directly generate ganglion mother cells instead of INPs; this ultimately results in fewer neural cells being produced.

It is equally important for type II neuroblasts to maintain their ability to generate self-renewing intermediate neural progenitors so that enough neural cells are produced. The transcription factor Pointed P1 is known to play an important role in this process (Zhu et al., 2011). However, the other proteins that type II neuroblasts might need in order to maintain their identity and generate intermediate neural progenitor cells were not known. Now, in *eLife*, researchers at the University of Michigan Medical School report that Trithorax, a protein that modifies histones, has a crucial role in these processes in *Drosophila* (Komori et al., 2014) while, independently, researchers at the State University of New York (SUNY) and Syracuse University report that a transcription factor called Buttonhead is also critically important (Xie et al., 2014).

The fate of cells can be controlled by a number of methods. For example, adding a methyl group to histone H3 at lysine 4 (H3K4) is known to be involved in controlling cell fate during development. Therefore, Cheng-Yu Lee and colleagues at Michigan, including Hideyuki Komori as the first author, tested whether Trithorax—a protein that adds methyl groups to H3K4—might also play a role in maintaining the identity of *Drosophila* neuroblasts (Komori et al., 2014). By using a combination of antibodies that recognize the different cell markers found on type I and type II neuroblast lineages, Komori et al. showed that Trithorax is necessary for maintaining the functional identity of type II neuroblasts as they proliferate. Thus, a significant reduction in the number of intermediate neural progenitors occurs in type II neuroblast lineages that have a defective version of the *trithorax* gene. These mutant type II neuroblasts instead transform into type I neuroblasts, which directly generate ganglion mother cells instead of intermediate neural progenitors (Figure 1).

To work in type II neuroblasts, Komori et al. found that Trithorax must have the ability to add methyl groups to histones. However, preventing Trithorax from working did not affect the overall H3K4 methylation pattern in type II neuroblasts, which suggests that Trithorax only regulates a few specific genes. Komori et al. identified a small number of genes specifically expressed in type II neuroblasts, including *buttonhead* and *pointed*, and using biochemical analysis found that Trithorax indeed binds directly to the transcription start site of both of these genes. In addition, removing working copies of the *buttonhead* gene from type II neuroblasts reduced the number of mature intermediate neural progenitors in the fly brain, and over-expressing *buttonhead* restored the ability of type II neuroblasts with mutant (less functional) *trithorax* genes to produce intermediate neural progenitor cells. Furthermore, mis-expressing *buttonhead* in type I neuroblasts caused these cells to behave more like type II neuroblasts and generate cells that resemble intermediate neural progenitor cells.

The role of *buttonhead* in the type II neuroblast lineages was confirmed and investigated further by Sijun Zhu of SUNY and colleagues, including Yonggang Xie as the first author (Xie et al., 2014). They also observed a marked reduction in the number of intermediate neural progenitor cells produced from *buttonhead* mutant type II neuroblasts, due to the premature differentiation of immature intermediate neural progenitors into ganglion mother cells. Moreover, Xie et al. showed that the Buttonhead protein most likely prevents this premature differentiation by suppressing the expression of a protein called Prospero that inhibits cell self-renewal and promotes cell cycle exit and differentiation. Xie et al. also provide evidence suggesting that in type II neuroblasts, Buttonhead can cooperate with the transcription factor Pointed P1 to specify the neuroblast function and promote the generation of intermediate neural progenitors (Figure 1).

Komori et al. and Xie et al. provide insight into how the identity of neural stem cells is maintained in the *Drosophila* brain. This work demonstrates that the histone modification protein Trithorax, together with its direct transcription factor targets Buttonhead and Pointed P1, is crucial for type II neuroblasts to maintain their identity and promotes the generation of intermediate neural progenitor cells.

In the future, it will be important to analyze how this identity maintenance program ensures normal brain development in *Drosophila* by interacting with a previously characterized program that restricts cell proliferation. Moreover, the mammalian versions of Trithorax (called SET/MLL) and Buttonhead (Sp8) play important roles in mammalian brain development. It will therefore also be important to determine if the Trithorax-dependent mechanisms identified in the fly also operate in the mammalian brain to maintain neural stem cell identity.
